# Italian Health Care Workers’ Knowledge, Attitudes, and Practices Regarding Human Papillomavirus Infection and Prevention

**DOI:** 10.3390/ijerph17155278

**Published:** 2020-07-22

**Authors:** Cecilia Trucchi, Vincenzo Restivo, Daniela Amicizia, Francesca Fortunato, Alessia Manca, Domenico Martinelli, Alfredo Montecucco, Maria Francesca Piazza, Rosa Prato, Valentino Tisa, Filippo Ansaldi, Giancarlo Icardi

**Affiliations:** 1 Azienda Ligure Sanitaria, 16121 Genoa, Italy; daniela.amicizia@unige.it (D.A.); mariafrancesca.piazza@regione.liguria.it (M.F.P.); filippo.ansaldi@unige.it (F.A.); 2 Planning and Prevention Unit, I.R.C.C.S. San Martino Hospital, 16132 Genoa, Italy; icardi@unige.it; 3 Department of Health Promotion Sciences, Maternal and Infant Care, Internal Medicine and Excellence Specialties (PROMISE), University of Palermo, 90127 Palermo, Italy; vincenzo.restivo@unipa.it; 4 Department of Health Sciences, University of Genoa, 16132 Genoa, Italy; alfredo.montecucco@edu.unige.it; 5 Department of Medical and Surgical Sciences, University of Foggia, 71121 Foggia, Italy; francesca.fortunato@unifg.it (F.F.); domenico.martinelli@unifg.it (D.M.); rosa.prato@unifg.it (R.P.); 6 Occupational Medicine Unit, I.R.C.C.S. San Martino Hospital, 16132 Genoa, Italy; alessia.manca@hsanmartino.it; 7 Department of Primary Care, Local Health Units 4, 16043 Chiavari, Italy; valentino.tisa@gmail.com

**Keywords:** HPV, knowledge, practice, attitude, awareness, healthcare workers, vaccine, immunization, prevention, sexually transmitted infection

## Abstract

*Objective:* To assess healthcare workers’ knowledge and attitudes about human papillomavirus (HPV) infection, related diseases, and prevention. *Methods:* A cross-sectional multicenter survey about HPV and its prevention, targeted to healthcare workers involved in HPV vaccine counseling, was performed from May 2017 to December 2018. *Results:* The overall median knowledge and attitude scores were 69.2% (25–75, *p* = 61.5–84.6) and 5 (25–75, *p* = 4–5), respectively. Both knowledge and attitudes statistically significantly differ between physicians and healthcare professions. The median propensity score before and after the educational intervention was stable and high, at 10 (25–75, *p* = 9–10). The predictors of statistically significantly high knowledge scores are to be a physician, general practitioner, or pediatrician, attending courses/congresses, and consulting technical product characteristics and scientific literature to obtain information about the HPV vaccine. Being a physician and consulting scientific literature to obtain information about the HPV vaccine were found also as predictors of statistically significantly different attitude scores among study participants. *Conclusions:* Although healthcare workers showed overall positive attitudes towards the relevance of HPV burden and prevention tools, demonstrated knowledge was largely suboptimal, particularly that shown by healthcare professions. Obtained results allow highlighting knowledge gaps, and thus improving counselling to HPV vaccine targets.

## 1. Introduction

Human papillomavirus (HPV) infection is still a rising public health widespread threat due to the morbidity, mortality, and costs related to benign and malignant diseases such as genital warts and cervical, vulvar, anal, penile, head, and neck cancers [1,2]. The major threat of HPV infections remains cervical cancer. Indeed, every year in Europe, about 60,000 women develop cervical cancer, and 25,800 die from the disease. Furthermore, cervical cancer has a younger age of onset compared to other adult cancers, and currently ranks as the second most frequent cancer in European women between 15 and 44 years of age [3].

The worldwide adopted strategy in cervical cancer prevention includes both HPV cervical screenings and vaccination. The HPV vaccine was demonstrated to be successful as a primary prevention intervention against cervical cancers, since its use has been associated with the reduction of HPV-related diseases, HPV-related cancers, and the prevalence of HPV genotypes with no reported serious adverse effects [4,5,6]. Despite the availability of efficacious, safe, and well-tolerated vaccines, coverage rates of HPV vaccination programs differ widely between countries and are suboptimal, even in countries with well-structured public health policies, such as Italy [7,8,9]. To address this criticism, in May 2017, the World Health Organization (WHO) highlighted the relevance of cervical cancer and other HPV-related diseases as a global public health threat, and conformed the recommendation to include HPV vaccines into national immunization programs as part of a coordinated and comprehensive strategy to prevent HPV-related diseases [10]. The successful implementation of primary prevention of HPV infection is based on an integrated approach that includes the analysis and management of organizational aspects of immunization campaigns and raising awareness among the general population and high-risk subjects [11,12,13,14,15,16]. This requires active involvement by the healthcare workers, who play a key role in promoting the HPV vaccination and its acceptability, since they can influence the whole health behavior of their patients. To be efficacious, healthcare workers need detailed key information on HPV and guidance on the best practice to communicate to their patients [17]. Furthermore, the information should vary radically depending on target. However, suboptimal knowledge and awareness among healthcare workers about the value of the HPV vaccine was found to be one of the major barriers to successful vaccination campaigns [18]. In Italy, several healthcare workers manage the promotion and administration of the HPV vaccine, such as pediatricians, general practitioners (GPs), gynecologists, and vaccination service healthcare workers. They have different target populations and need to build up a promotion campaign that deals with the same theme but adopts different language.

The present study aimed at evaluating the professional characteristics, the knowledge, the opinions, and estimating the training needs of Italian multi-professional health workers involved in the vaccination counselling/choice, and in the management of the prevention interventions related to the HPV infection, pathologies, and vaccination.

## 2. Materials and Methods 

### 2.1. Study Design and Participants

A cross-sectional multicenter survey about HPV and its prevention, targeted to healthcare workers, was performed from May 2017 to December 2018. The target population included healthcare workers such as physicians, nurses, and healthcare assistants who can recommend the HPV vaccine during their activity. Recruitment of the study participants took place at the main Liguria Hospital, San Martino Hospital in Genoa, the main Ligurian vaccination centers, and across the Ligurian and Apulian network of primary care. After the administration of an informed consent reporting the study aims and objectives, the participant’s right of refusal, and the possibility to terminate the participation in the study at any time and without disadvantage, participants filled a written or web-based anonymous survey. The sample size was calculated considering the number of subjects belonging to the target study healthcare personnel and the available evidences about the HPV vaccine acceptability, fixing the confidence interval at 95%, with a margin of error of (+/-) 2.5%, and adding a quote of 5%, in consideration of the possible drop-outs and missed answers. 

### 2.2. Data Collection and Questionnaire

Ad hoc anonymous questionnaires addressed to healthcare workers were developed. They were self-administered using the Google Drive platform that automatically populates and saves digital responses into a secure Microsoft Excel database, protecting participant confidentiality throughout the surveying process. Participants could also fill out two written informed consent and questionnaires characterized by the same structure of those administered online. The first questionnaire was set in two sections, the first investigated demographics (age and sex), profession, and, for physicians, type of specialty (i.e., public health, infectious diseases, gynecology, otolaryngology, pediatrics, psychiatry, urology, oncology, primary care), number of patients for primary care specialists, professional experience (expressed in year), source used for seeking information on the HPV vaccine (courses, congresses, colleagues, institutional websites, scientific societies websites, summary of product characteristics, national/international scientific literature, blog, and others). The enrolled subjects were also asked if they usually recommend HPV vaccines on the occasion of counselling to pre-adolescents of both sexes and subjects at risk, such as those with HIV, homosexuals, etc. The first section also included a 10-point Likert scale ranking the intention to promote the HPV vaccine. The second section included thirteen questions on the knowledge on HPV infection, its related diseases, and vaccine. The second questionnaire assessed participants’ attitudes using nine statements with a 5-point Likert scale for each one (5 points: strongly agree; 4 points: agree; 3 points: neutral; 2 points: disagree; 1 point: strongly disagree). It also included a 10-point Likert scale ranking the intention to promote the HPV vaccine after reading some information on HPV infection, related diseases, and vaccine, included at the beginning of the second questionnaire (self-learning). 

### 2.3. Statistical Analyses

Statistical analysis was performed by means of JMP software (version 13.0.0, SAS Institute Inc., Cary, NC, USA) and Excel version 2016 (Microsoft Corporation, Redmond, WA, US) after generating data from the written questionnaires and Google Drive (Google, Mountain View, CA, USA). Median and 25–75 percentiles for continuous variables, frequencies, and percentages for categorical variables were calculated. Based on the participants’ responses, the “knowledge”, “attitude”, and “intention” scores were calculated. The “knowledge” score summarizes the percentage of correct answers the healthcare worker gave. For each knowledge statement, one point was given for a correct answer (true or false), and zero points if they selected a wrong answer or “do not know”. The total points were added for every participant, and a percentage was calculated (number of correct answers/number of knowledge statements × 100). The higher the knowledge score, the more knowledgeable the participant is regarding HPV infection, its related diseases, and prevention. The attitude score was obtained by calculating the average of the healthcare workers’ responses to the statements on a five-point Likert scale. The median of the total points was calculated for every participant obtaining an attitude score ranging from 1.0 to 5.0. The closer the attitude score to 5, the more positive the participant’s attitude. Finally, the “intention” score reflects the intention to promote the HPV vaccine. The “intention” score was estimated both at the beginning and at the end of the survey, when the information about HPV infection and vaccination had been given. The score is on a scale from 1 to 10, where 10 reflects the highest propensity to promote the HPV vaccine. The possible association of baseline characteristics of young adults and at-risk subjects with knowledge and attitude scores was tested through univariable logistic regression. A *p*-value less than 0.05 was considered significant throughout the study. Multivariate analysis was performed to identify the predictors of having high knowledge score or positive attitude score towards HPV vaccination. Only covariates that showed a significance level of *p*-value < 0.1 in the univariate analysis were included in the multivariable regression model, using a backward stepwise algorithm. Given the high number of potential independent variables, a backward stepwise algorithm was used to identify the best-fitting subset of variables to use in the final regression model. In particular, Akaike’s information criterion (AIC) was used to assess the models’ fit, and the model with the lowest AIC was selected for the multivariable analysis. β-coefficients (95% CI) and *p*-value were estimated. 

### 2.4. Ethics Approval and Consent to Participate

In complying with the highest ethical standards, participants were informed that their participation was entirely voluntary and that they could withdraw at any moment. All data obtained through their participation was kept strictly confidential among the research team. In addition, an informed consent was obtained after explaining the nature of the study and its possible consequences. The study protocol was approved by the Liguria Regional Ethics Committee (P.R. 162REG2017) for the study coordination center.

## 3. Results

### 3.1. Participants’ Characteristics

A total of 1410 subjects (53.6% females and 41.5% males) participated in the survey (Table 1). The baseline characteristics of study participants are shown in Table 1. The median age was 33 (28–57) years. Considering the profession, 681 (48.3%) were physician specialists in public health, infectious diseases, gynecology, oncology, dermatology, urology, and otorhinolaryngoiatry, 398 (28.2%) were GP or pediatrician, and 259 (18.4%) belonged to healthcare professions such as nurse, health assistant, and obstetrician. With regard to the source of information about the HPV vaccine, most (57.6%) declared they were informed from courses and congresses, 42.8% from national/international scientific literature, 30.5% from colleagues, and 23.3% use official websites such as Ministry of Health, Epicentro, and VaccinarSi. Scientific societies’ websites are also used by 14.8% of subjects, while 12.7% usually surf the internet to find information on HPV. Summaries of product characteristics of vaccine, regional authority/local health unit websites, and blogs are consulted by 19.3%, 12.7% and 0.2%, respectively. A large number of physicians/healthcare workers (1226, 87%) recommend the HPV vaccine to pre-adolescents; 6.5% stated the opposite; and 6.5% chose not to express opinion. Regarding recommendation of the HPV vaccine to subjects at risk, 82.6% (1165) answered affirmatively, 9.8% (138) answered in the negative, and 7.6% preferred not to declare their approach.

### 3.2. Knowledge of Human Papillomavirus Infection

Table S1 reports the correct responses to individual knowledge of HPV items among all professional groups. The overall median knowledge score was 69.2% (25–75, *p* = 61.5–84.6). With regard to the occurrence of signs and symptoms related to HPV infection, the majority of healthcare workers were aware that the majority of HPV-infected subjects are asymptomatic and can transmit the infection. Furthermore, only 65.5% knew that a negative pap test does not mean the patient is uninfected. The existence of various genotypes of HPV was known by 82.4% of participating healthcare workers. Declared knowledge about HPV-related malignant diseases showed that 74.6% of healthcare workers are aware of the role of HPV in causing anal cancer, and only 31.1% knew that HPV is the necessary condition for developing all cervical cancers. With regard to other HPV-related lesions, only 43.4% and 73.1% knew that HPV 6 and HPV 11 are not precursors of cervical cancer, and that HPV does not cause genital herpes, respectively; furthermore, 89% correctly identified HPV as a causative agent of genital warts. Finally, the correct HPV vaccine schedule recommended for pre-adolescents was known only by 73.7% of subjects; less than half of healthcare workers were aware that the available HPV vaccine is not live attenuated, and about 37% knew that testing HPV infection before vaccine administration is not necessary. Statistically significant differences among the knowledge shown by participating healthcare workers were observed in all the statements, with medical doctors showing better knowledge than other healthcare professions (e.g., nurses, health assistants, and obstetricians). 

### 3.3. Attitudes of Health Workers toward Human Papillomavirus Infections, Related Diseases, and Prevention Statements

Table S2 reports the attitudes of healthcare workers toward HPV infections, related diseases, and prevention statements. The overall median attitude score was as high as 5 (25–75, *p* = 4–5). A total of 87.7% of healthcare workers agree/strongly agree that HPV is the main cause of genital warts. With regard to attitudes towards HPV vaccine performances, 84.1%, 92.6%, and 92.1% strongly agree/agree that the HPV vaccine prevents the infections caused by the more common genotypes, that it plays the main role in primary prevention of cervical cancer, and that it represents the main prevention tool together with screening, respectively. Furthermore, 89% consider the HPV vaccine safe and well-tolerated. With regard to vaccine recommendations, almost all would get their own pre-adolescent son/daughter vaccinated against HPV, and 87.1% were favorable in making the vaccine compulsory for them. The vaccine choice process was considered largely influenced by pre-adolescents’ parents by 73.3% of participant healthcare workers. Finally, only 60.8% considered the co-payment cost affordable and balanced with the offered benefits.

The attitudes also statistically significantly differ between healthcare workers and medical doctors, showing more positive attitudes than other healthcare professions (e.g., nurses, health assistants, and obstetricians). In particular, medical doctors strongly agreed to all the proposed sentences more frequently than other healthcare professions. 

### 3.4. Knowledge and Attitude Scores 

In Table S3, the knowledge and attitude scores stratified by health workers characteristics are shown. Considering participants’ age, the median knowledge score increases with age, but no statistical differences were observed. The same finding was obtained when comparing males and females. A statistically significant difference was observed, instead, between the knowledge scores shown by medical doctors and health professions; both physicians, GPs, and pediatricians, in fact, demonstrated better knowledge than participants of health professions. No relevant differences in knowledge scores were observed considering the number of patients assisted by each GP/pediatrician and the duration of professional experience at the moment of the survey. Among declared sources of information about the HPV vaccine, courses, congresses, scientific societies’ websites, technical product characteristics, and scientific literature were statistically significantly related to higher knowledge scores. Finally, with regard to the declared activity of recommending the HPV vaccine to target subjects, statistically significantly better knowledge was shown by healthcare workers who recommend it to pre-adolescents; healthcare workers who declared recommending the HPV vaccine to at-risk subjects, instead, showed lower knowledge scores, although they were not statistically significantly different. 

The analysis of attitude scores demonstrated high median values for all considered characteristics, with statistically significant differences observed stratifying study population by profession and among those who reported scientific societies’ websites as a source of information about HPV vaccine.

### 3.5. Propensity Toward Human Papillomavirus Vaccination

The median propensity score before and after the educational intervention was stable and high, at 10 (25–75, *p* = 9–10).

### 3.6. Multivariate Analysis

The results of the multivariate analysis predicting high knowledge and attitude scores are presented in Table 2 and Table 3, respectively. The predictors of statistically significantly high knowledge scores are to be a physician, GP, or pediatrician, attending courses/congresses, and consulting technical product characteristics and scientific literature to obtain information about the HPV vaccine. Being a physician and consulting scientific literature to obtain information about the HPV vaccine were found also as predictors of statistically significantly different attitude scores among study participants. 

## 4. Discussion

Anti-HPV vaccination and cervical cancer screening tests represent an effective and safe immunization tool and a key secondary preventative strategy to reduce HPV-related burden, respectively. Despite the availability of high-performing HPV vaccines, national vaccination coverage of such a consolidated target as girls remains suboptimal in European countries, including Italy [9,19]. 

Healthcare providers play a key role in counseling parents to immunize their children, since they are recognized as authoritative and highly trusted sources of information [20]. Indeed, provider recommendation is the strongest predictor of HPV vaccine compliance by the main targets (e.g., adolescents and at-risk subjects, such as homo- and bisexuals) [21,22,23,24,25,26,27,28,29,30,31]. Thus, it is essential to investigate the levels of HPV-related knowledge and attitudes among healthcare workers involved in HPV vaccine choice (such as public health officers, gynecologists, and primary care physicians) towards anti-HPV vaccination, to improve their awareness and to enhance pro-HPV vaccination attitudes among physicians. 

This study was performed in order to evaluate knowledge and attitudes of Italian health workers of HPV burden and prevention, and their correlation with professional characteristics.

Findings of the present study indicated that only 73.3% of participants are aware they play a relevant role in the HPV vaccine choice process faced by pre-adolescents’ parents and target subjects. This lack of consciousness is of particular concern, since it could contribute to failing the achievement of the vaccine coverage goal.

As found in previous studies, the majority of interviewed Italian healthcare workers declared they routinely recommend the HPV vaccine to pre-adolescents and subjects at risk (87% and 82.6%, respectively), and recognize the benefits of the HPV vaccine in the prevention of cervical cancer, combined with screening, and the infection caused by the most common HPV genotypes [32,33].

Protection against cervical cancer, genital warts, and the infections caused by the more common genotypes were the most commonly cited benefits of HPV vaccination, whereas worse attitudes were reported towards the cost-benefits rate.

Differently from previous studies [32], healthcare workers’ sex differences were not found in attitude score regarding HPV burden and prevention. This observation probably demonstrates the overcoming of the conception that HPV is a female-specific disease [34]. 

Although most healthcare workers supported HPV vaccination and showed positive attitudes related to HPV, the study highlighted several knowledge gaps, as observed previously in other industrialized countries, including Italy [35,36,37,38,39]. 

Suboptimal knowledge was shown in both HPV-related diseases impact (e.g., HPV was recognized as a necessary condition for developing cervical cancer only by 31.1% of participants) and prevention tools (e.g., 42.8%, 37.1%, and 65.5% of participants were aware that available vaccines against HPV are not live attenuated, knew that it is not necessary to test HPV infection before vaccine administration, and that a negative pap test does not mean the patient is uninfected, respectively). Nevertheless, overall knowledge shown by the interviewed sample was higher than that demonstrated by previous published Italian data obtained by general practitioners, highlighting an improvement in professional education during the last years [39]. 

Health professionals need to feel prepared and to be supported in their role of eliciting perceptions and addressing concerns about HPV, offering the vaccine, and reaching a shared decision with their patients. Consistent with previous studies, our results show that health professionals need to access multiple sources of information, such as course/congresses, scientific literature, and technical product characteristics, that significantly contribute to their knowledge score [33,40,41,42,43]. With regard to improving HPV education, previous studies showed also that the web-based continued medical education in the context of cervical cancer screening has favorable results, particularly in terms of increasing knowledge levels and enhancing the adoption of clinical guidelines [44]. The use of e-learning within medical education, in fact, has been shown to be a useful supplement to traditional teaching methods, because it allows learner-centered education and overcomes geographical boundaries [45,46]. Furthermore, Berenson et al. conducted a study involving 427 healthcare providers in the United States and found that the HPV knowledge score of providers increased after attending an educational lecture [47].

Since the propensity score towards HPV vaccination was found to be high even before the educational intervention, our study did not allow us to demonstrate its efficacy. Nevertheless, this result confirmed the very positive attitudes of participant healthcare workers.

Of interest, as observed in a previous study [48], the length of professional experience does not correlate with HPV knowledge score. This observation suggests that the awareness of HPV burden is not influenced by clinical experience, and that wide HPV education is needed. 

Furthermore, the acceptance of the HPV vaccine is influenced by a wide range of factors, which include not only public and healthcare workers’ knowledge and attitudes towards the vaccine, but also health information communication techniques [49]. 

Indeed, a previous study demonstrated that in some cases a professional’s own knowledge will not be enough to satisfy patients’ informational needs and address their concerns [50]. Thus, health professionals need to be supported through several educational initiatives that will allow them to communicate health information efficiently, taking into account patients’ perspectives [49]. 

In order to support health professionals in HPV vaccine counseling, various interventions were investigated. 

In particular, a study by McSherry et al. aimed at developing a theory-based intervention to support primary care practitioners in their HPV-related practices confirmed the need for an intervention to support primary care practitioners around HPV, and found that the Theoretical Domains Framework (TDF) proved valuable in analyzing qualitative data collected, using a topic guide and understanding HPV-related clinical behaviors [37]. 

Another project, conducted by Giguere et al., investigated the use of decision boxes to prepare the clinician to communicate the risks and benefits of the available options to the patient, and to explore patients’ values and preferences in management options to support a shared decision-making process [51]. 

Furthermore, a structured approach to communicating vaccination information according to patients’ readiness to vaccinate was investigated as a support tool by Leask et al. [52].

The strength of our study is the comprehensive approach, which considers multiple disciplines involved in the prevention, screening, and treatment of the HPV infection and related diseases, such as cervical cancer, both in the fields of research and practice. Of note, significant differences in knowledge and attitudes were observed among physicians, GP/pediatricians, and other health professionals (i.e., nurses, health assistants, obstetricians). This is of particular concern, since HPV knowledge in nurses has been identified as an important precursor for a positive attitude towards the vaccine [53,54], and scientific authorities, such as the Centers for Disease Control and Prevention, have created educational material to support them in counseling adolescents and their parents [55,56]. Nevertheless, the investigated sample could be not fully representative of Italian healthcare professionals, since only the most relevant medical specialties and professions were investigated. Future research could also involve community pharmacists, who have frequent contact with the public, and frequently represent the first interlocutor of people to debate health related topics, including infection prevention. 

Another limit is represented by the involvement of Northern and Southern Italian regions, so the national representativeness could be suboptimal.

## 5. Conclusions

Although participant healthcare workers showed overall positive attitudes towards the relevance of HPV burden and prevention tools, demonstrated knowledge was largely suboptimal, particularly that shown by nurses, health assistants, and obstetricians.

Obtained results allow addressing educational intervention in terms of target, arguments, and modalities. In particular, health professions with higher knowledge needs, items with low proportion of correct responses, and preferred sources of information were analyzed and deepened. This could increase HPV knowledge and improve the capacity of providers to offer HPV preventive counselling and vaccination to HPV vaccine targets.

## Figures and Tables

**Table 1:** Baseline characteristics of study participants.

Variables	*n (%)*	*Median (25–75p)*
**Age**		33 (28–57)
**Sex**		
Female	756 (53.6)	
Male	585 (41.5)	
NR	69 (4.9)	
**Profession**		
Physician (Public health, Infectious diseases, Gynecology, Oncology, Dermatology Urology, Otorynolaringoiatry specialists)	681 (48.3)	
General practitioners/ Pediatricians	398 (28.2)	
Nurse, Health assistant, Obstetrician	259 (18.4)	
NR	72 (5.1)	
**Patients of GP/Pediatrician**		931 (800–1338)
NR		
**Professional experience (years)**		11 (3–30)
NR		
**Source of information about HPV vaccine**		
Course/Congress	812 (57.6)	
Colleagues	430 (30.5)	
Ministry of Health, Epicentro, VaccinarSi websites	329 (23.3)	
Scientific societies websites	209 (14.8)	
Regional authority/Local Health Unit websites	179 (12.7)	
Summary of Product Characteristics of vaccine	272 (19.3)	
National/international Scientific literature	603 (42.8)	
Blog	3 (0.2)	
**Recommendation of HPV vaccine to pre-adolescents**		
Yes	1226 (87.0)	
No	92 (6.5)	
NR	92 (6.5)	
**Recommendation of HPV vaccine to subjects at risk**		
Yes	1165 (82.6)	
No	138 (9.8)	
NR	108 (7.6)	
**Total**	1410 (100)	

NR: non-responded, GP: general practitioners, HPV: human papillomavirus.

**Table 2:** Multivariate analysis for predicting knowledge score.

Characteristics	β-Coefficient (95% C.I.)	95% C.I. Lower Limit	95% C.I. Upper Limit	*p*-Value
Physician, GP/Pediatricians	7.3849	5.3742	9.3958	<0.0001
Courses/Congresses	3.1588	1.5298	4.7879	0.0002
Technical product characteristics	3.1631	1.1501	5.1762	0.0021
Scientific literature	2.3701	0.7525	3.9876	0.0042

C.I.: Confidence Interval, GP: general practitioners.

**Table 3:** Multivariate analysis for predicting attitude score.

Characteristics	β-Coefficient (95% C.I.)	95% C.I. Lower Limit	95% C.I. Upper Limit	*p*-Value
Physician	0.0635	0.0162	0.1109	0.0087
Scientific literature	0.0946	0.0466	0.1425	0.0001

C.I.: Confidence Interval.

## Supplementary Materials

The following are available online at https://www.mdpi.com/1660-4601/17/15/5278/s1, Table S1: Correct responses (*n*/%) to individual knowledge of HPV items among all professional groups (*n* = 1410); Table S2: Attitudes of health workers (*n*/%) toward HPV infections, related diseases, and prevention statements; Table S3: Knowledge and attitude scores stratified by healthcare workers characteristics.
