# Supplementary material for: Italian Health Care Workers’ Knowledge, Attitudes, and Practices Regarding Human Papillomavirus Infection and Prevention
Source: Int J Environ Res Public Health. 2020 Jul 22;17(15):5278. doi: 10.3390/ijerph17155278 (PMC7432815; doi:10.3390/ijerph17155278)
Supplement: Supplementary file 1 [file ijerph-17-05278-s001.pdf]

Table S1. Correct responses (n/%) to individual knowledge of HPV items among all professional groups (n = 1,410).

Knowledge statement	Response	Overall (n=1,410)	Physician (n=681)	GP/ Pediatricians (n=397)	Nurse, Health assistant, Obstetrician (n=260)	NR (n=72)	p-value
Type of cancer HPV-related: anal cancer	true	1052 (74.6)	591 (86.8)	343 (86.5)	116 (44.8)	0	<0.0001
HPV is the necessary condition for developing 100% of cervical cancer	true	438 (31.1)	236 (34.7)	134 (33.8)	69 (26.4)	0	<0.0001
Genital warts caused by HPV 6 and HPV 11 are precursors of cervical cancer	false	612 (43.4)	349 (51.3)	182 (45.9)	78 (29.9)	3 (4.2)	<0.0001
The majority of HPV infections are asymptomatic	true	1228 (87.1)	645 (94.7)	355 (89.5)	224 (86.2)	3 (4.2)	<0.0001
HPV can cause genital herpes	false	1031 (73.1)	601 (88.2)	287 (72.2)	140 (54.0)	3 (4.2)	<0.0001
HPV can cause genital warts	true	1255 (89.0)	657 (96.5)	373 (94.0)	221 (85.1)	3 (4.2)	<0.0001
The majority of infected subjects are asymptomatic	true	1210 (85.8)	651 (95.6)	352 (88.7)	203 (78.2)	3 (4.2)	<0.0001
HPV infections are caused by the same type of virus	false	1162 (82.4)	648 (95.2)	331 (83.5)	179 (69.0)	3 (4.2)	<0.0001
HPV infection can only be transmitted if the carrier is symptomatic	false	1273 (90.3)	669 (98.3)	382 (96.2)	218 (83.9)	3 (4.2)	<0.0001
Negative Pap test means being uninfected	false	924 (65.5)	499 (73.3)	272 (68.4)	152 (58.6)	0	<0.0001
HPV vaccine schedule for pre-adolescents subjects includes the administration of two doses 6 months apart	true	1039 (73.7)	520 (76.3)	343 (86.5)	173 (66.7)	3 (4.2)	<0.0001
It is necessary to test HPV infection before vaccine administration	false	523 (37.1)	197 (29.0)	251 (63.2)	75 (28.7)	0	<0.0001
HPV vaccine is live attenuated	false	603 (42.8)	248 (36.4)	257 (64.7)	96 (36.8)	3 (4.2)	<0.0001

Table S2. Attitudes of health workers (n/%) toward HPV infections, related diseases, and prevention statements.

		Attitude Statement								
		HPV is the main cause of genital warts	HPV vaccine plays the main role in primary prevention of cervical cancer	HPV vaccine is useful because it prevents the infections caused by the more common genotypes	HPV vaccine is safe and well tolerated	HPV vaccine is the main prevention tool together with screening	If I had a pre-adolescent son/daughter I get him/her vaccinated against HPV	I agree to make the HPV vaccine compulsory for boys and girls before the sexual debut and at risk subjects	The HPV vaccine co-payment cost is affordable and balances the offered benefits	I think to play a relevant role in HPV vaccine choice process by pre-adolescents' parents and target subjects
**Strongly Agree**	**Overall (n=1410)**	702 (49.8)	1019 (72.3)	729 (51.7)	729 (51.7)	991 (70.3)	1060 (75.2)	873 (61.9)	367 (26.0)	451 (32.0)
	**Physician (n=681)**	406 (59.7)	534 (78.4)	370 (54.4)	391 (57.5)	519 (76.32)	534 (78.4)	433 (63.6)	185 (27.1)	197 (29.0)
	**Primary care (n=397)**	176 (44.4)	278 (69.9)	197 (49.5)	179 (45.1)	269 (67.7)	307 (77.4)	251 (63.2)	110 (27.8)	170 (42.8)
	**Healthcare professions (n=260)**	90 (34.5)	158 (60.8)	131 (50.5)	128 (49.4)	158 (60.8)	170 (65.5)	143 (55.1)	51 (19.5)	69 (26.4)
	**NR (n=72)**	30 (41.7)	48 (66.6)	30 (41.7)	30 (41.7)	45 (62.5)	48 (66.7)	45 (62.5)	21 (29.2)	15 (20.8)
**Agree**	**Overall (n=1410)**	535 (37.9)	286 (20.3)	457 (32.4)	526 (37.3)	307 (21.8)	245 (17.4)	355 (25.2)	491 (34.8)	582 (41.3)
	**Physician (n=681)**	212 (31.1)	90 (13.2)	179 (26.3)	212 (31.1)	93 (13.7)	90 (13.2)	146 (21.5)	221 (32.5)	275 (40.4)
	**Primary care (n=397)**	197 (49.6)	116 (29.3)	182 (45.9)	194 (48.9)	122 (30.8)	81 (20.3)	128 (32.2)	138 (34.6)	185 (46.6)
	**Healthcare professions (n=260)**	107 (41.4)	63 (24.1)	69 (26.4)	90 (34.5)	66 (25.3)	57 (21.8)	66 (25.3)	95 (36.8)	92 (35.6)
	**NR (n=72)**	18 (25)	18 (25)	27 (37.5)	30 (41.7)	27 (37.5)	18 (25)	15 (20.8)	36 (50.0)	30 (41.7)
**Neutral**	**Overall (n=1410)**	72 (5.1)	18 (1.3)	86 (6.1)	77 (5.4)	28 (2)	21 (1.5)	86 (6.1)	379 (26.9)	266 (18.9)
	**Physician (n=681)**	12 (1.8)	12 (1.8)	39 (5.7)	33 (4.8)	18 (2.7)	12 (1.8)	45 (6.6)	179 (26.3)	137 (20.1)
	**Primary care (n=397)**	12 (3.0)	0	15 (3.8)	24 (6.0)	6 (1.5)	0	15 (3.8)	119 (30.1)	39 (9.8)
	**Healthcare professions (n=260)**	30 (11.5)	3 (1.2)	18 (6.9)	9 (3.5)	3 (1.2)	3 (1.2)	15 (5.8)	66 (25.3)	66 (25.3)
	**NR (n=72)**	18 (25)	3 (4.2)	15 (20.8)	12 (16.7)	0	6 (8.3)	12 (16.7)	15 (20.8)	24 (33.3)
**Disagree**	**Overall (n=1410)**	21 (1.5)	6 (0.4)	42 (3.0)	0	3 (0.2)	6 (0.4)	18 (1.3)	83 (5.9)	30 (2.1)
	**Physician (n=681)**	3 (0.4)	0	36 (5.3)	0	3 (0.4)	0	9 (1.3)	45 (6.6)	24 (3.5)
	**Primary care (n=397)**	12 (3.0)	3 (0.8)	3 (0.8)	0	0	6 (1.5)	3 (0.8)	30 (7.5)	3 (0.8)
	**Healthcare professions (n=260)**	0	3 (1.2)	3 (1.2)	0	0	0	6 (2.3)	9 (3.5)	3 (1.2)
	**NR (n=72)**	6 (8.3)	0	0	0	0	0	0	0	0
**Strongly Disagree**	**Overall (n=1410)**	0	3 (0.2)	13 (0.9)	0	6 (0.4)	3 (0.2)	3 (0.2)	0	3 (0.2)
	**Physician (n=681)**	0	0	9 (1.3)	0	3 (0.4)	0	3 (0.4)	0	3 (0.4)
	**Primary care (n=397)**	0	0	0	0	0	3 (0.8)	0	0	0
	**Healthcare professions (n=260)**	0	3 (1.2)	3 (1.2)	0	3 (1.2)	0	0	0	0
	**NR (n=72)**	0	0	0	0	0	0	0	0	0
**NR**	**Overall (n=1410)**	80 (5.7)	78 (5.5)	83 (5.9)	78 (5.5)	75 (5.3)	75 (5.3)	75 (5.3)	90 (6.4)	78 (5.5)
	**Physician (n=681)**	48 (7.0)	45 (6.6)	48 (7.0)	45 (6.6)	45 (6.6)	45 (6.6)	45 (6.6)	51 (7.5)	45 (6.6)
	**Primary care (n=397)**	0	0	0	0	0	0	0	0	0
	**Healthcare professions (n=260)**	33 (12.6)	30 (11.5)	36 (13.8)	33 (12.6)	30 (11.5)	30 (11.5)	30 (11.5)	39 (14.9)	30 (11.5)
	**NR (n=72)**	0	3 (4.2)	0	0	0	0	0	0	3 (4.2)
**p-value**		<0.0001	0.0006	<0.0001	<0.0001	0.0011	0.0006	0.0200	0.0049	0.0008

Table S3. Knowledge and attitude scores stratified by healthcare workers characteristics.

	Knowledge score (median, 25-75p)	p-value	Attitude score (median, 25-75p)	p-value
**Age (years)**		0.4129		0.2833
<35	69.2 (61.5-84.6)		5 (4-5)	
35-50	69.2 (50-83.3)		5 (4-5)	
>50	76.9 (61.5-84.6)		5 (4-5)	
**Sex**		0.6241		0.0534
Female	69.2 (61.5-84.6)		5 (4-5)	
Male	76.9 (61.5-84.6)		5 (4-5)	
**Profession**		0.0010		0.0023
Physician	76.9 (66.7-84.6)		5 (4-5)	
GP/Pediatricians	76.9 (69.2-84.6)		5 (4-5)	
Health professions	61.5 (38.5-76.9)		5 (4-5)	
**Patients of GP/Pediatricians (n)**		0.1007		0.5649
<800	76.9 (76.9-88.5)		5 (4-5)	
800-1200	76.9 (69.2-84.6)		5 (4-5)	
>1200	76.9 (69.2-84.6)		5 (4-5)	
**Professional experience (years)**		0.9064		0.3184
<15	76.9 (61.5-84.6)		5 (4-5)	
15-30	76.9 (69.2-84.6)		5 (4-5)	
>30	76.9 (61.5-84.6)		5 (4-5)	
**Source of information about HPV vaccine**				
Courses/Congresses		<0.0001		0.1576
Yes	76.9 (69.2-84.6)		5 (4-5)	
No	69.2 (57.1-80.6)		5 (4-5)	
Colleagues		0.6363		0.1692
Yes	76.9 (61.5-84.6)		5 (4-5)	
No	69.2 (61.5-84.6)		5 (4-5)	
Ministry of health, Epicentro by National Health Institute, "VaccinarSi" by Hygiene National Society websites		0.2465		0.1756
Yes	76.9 (68.6-92.3)		5 (4-5)	
No	69.2 (61.5-84.6)		5 (4-5)	
Scientific societies websites		0.0171		0.0082
Yes	76.9 (69.2-91.8)		5 (4-5)	
No	69.2 (61.5-84.6)		5 (4-5)	
Regional and Local Health Authorities websites		0.5499		0.7661
Yes	76.9 (63.5-84.6)		5 (4-5)	
No	69.2 (61.5-84.6)		5 (4-5)	
Technical product characteristics		0.0058		0.0363
Yes	76.9 (69.2-92.3)		5 (4-5)	
No	69.2 (61.5-84.6)		5 (4-5)	
Scientific literature		0.0281		<0.0001
Yes	76.9 (69.2-84.6)		5 (4-5)	
No	69.2 (53.8-84.6)		5 (4-5)	
Recommendation of HPV vaccine to target subjects		0.0415		0.0504
Yes	76.9 (61.5-84.6)		5 (4-5)	
No	69.2 (53.8-76.9)		5 (4-5)	
Recommendation of HPV vaccine to at-risk subjects		0.5324		0.7833
Yes	69.2 (61.5-84.6)		5 (4-5)	
No	76.9 (67.3-76.9)		5 (4-5)	
